# Three-Dimensional Planar Alignment of Nematic Liquid Crystal by Direct Laser Writing of Nanogratings

**DOI:** 10.1021/acsphotonics.5c01342

**Published:** 2025-10-16

**Authors:** Uroš Jagodič, Jaka Pišljar, Andreja Jelen, Miha Škarabot, Igor Muševič

**Affiliations:** † Condensed Matter Department, 61790 Jožef Stefan Institute, Ljubljana SI-1000, Slovenia; ‡ Faculty of Mathematics and Physics, University of Ljubljana, Ljubljana SI-1000, Slovenia

**Keywords:** 3D printing, liquid crystals, alignment layer, direct laser writing, displays, microphotonic elements

## Abstract

We demonstrate a new method of aligning liquid crystals along polymer surfaces that are printed vertical to the focal plane using direct laser writing. The method is based on nanogrooves that are imprinted into surfaces of polymer structures and provide robust, reliable, repeatable, and well-controlled alignment patterns. Our results demonstrate that the anchoring strength of a liquid crystal on printed nanogratings is comparable to that of conventional polyimide layers. The advantages are at least 2-fold. First, we can print large vertical areas of well-defined patterns of nanogrooves with uniform anchoring strength, and, second, we can control the azimuthal anchoring strength by adjusting the amplitude and the periodicity of nanogrooves. Printing of alignment nanogrooves on tilted, curved and surfaces of arbitrary shape could be realized using printing protocols based on the principle shown here with potential applications in emerging microphotonic devices based on liquid crystals.

## Graphical abstract

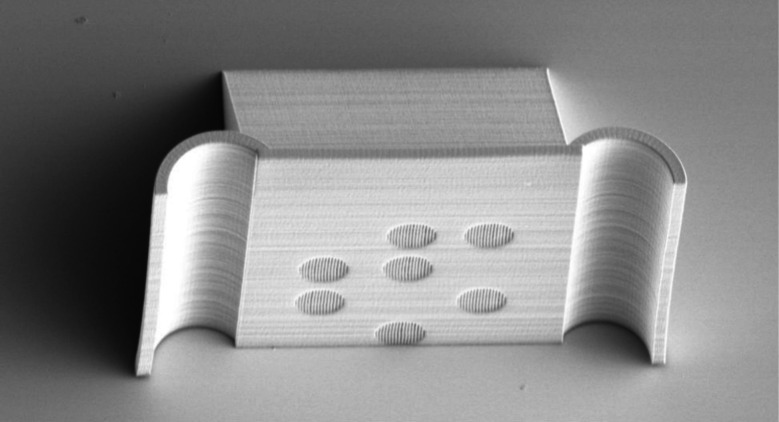

## 1 Introduction

It was shown by Berreman^1^ that mechanically produced microgrooves on a surface of a material, can very efficiently align nematic liquid crystals (NLCs) over macroscopic lengths, providing the NLC molecules locally align with their long axis parallel to the surface of the material. This is because the NLC is in a state of minimum of free energy, when the molecules are all aligned along the microgrooves. Any departure of molecular orientation from this direction results in elastic distortion of the NLC that increases the free energy of the NLC. Various techniques have been used to produce well-defined patterns of micro/nanogrooves to align the NLCs.^2,3^ The method of scratching a smooth polymer surface by the stylus of an AFM was used to create surface patterns,^4–6^ bistable and multistable surfaces^7–9^ for NLCs. Microtextured surfaces for LC alignment can also be lithographically fabricated using Digital Micromirror Devices^10,11^ or plasmonic photopatterning.^12^

The advent of the two photon polymerization technique, also called the Direct Laser Writing (DLW), has made it possible to directly write surface 2D patterns of arbitrary complexity and nanometer accuracy. Here, an infrared beam of a Gaussian profile is tightly focused in a photosensitive resin, and the intensity of the beam is set above the level for two-photon polymerization. This polymerizes the resin in a tiny voxel with ∼120 nm diameter in the plane perpendicular to the optical axis of the objective and ∼300 nm in the longitudinal direction. By moving the laser beam, objects are printed line-by-line at a given hatching distance (H) and then layer by layer at a given slicing distance (S), as illustrated in Figure 1. DLW has been used to fabricate thin polymer layers on glass to induce orientational alignment of LCs with an azimuthal anchoring energy of ∼8 × 10^–6^ J/m^2^, which is sufficient for most NLC electro-optical applications.^13–15^ DLW printed nanogratings also show good alignment of water-based liquid crystals,^16,17^ which are difficult to align otherwise. Complex alignment layers have been achieved by DLW in photosensitive materials to control light emission patterns.^18^ DLW has been used to fabricate 3D switchable diffractive optical elements,^19^ 3D printed photoresponsive materials,^20^ and 3D microdevice using liquid crystals.^21^

**Figure 1:**
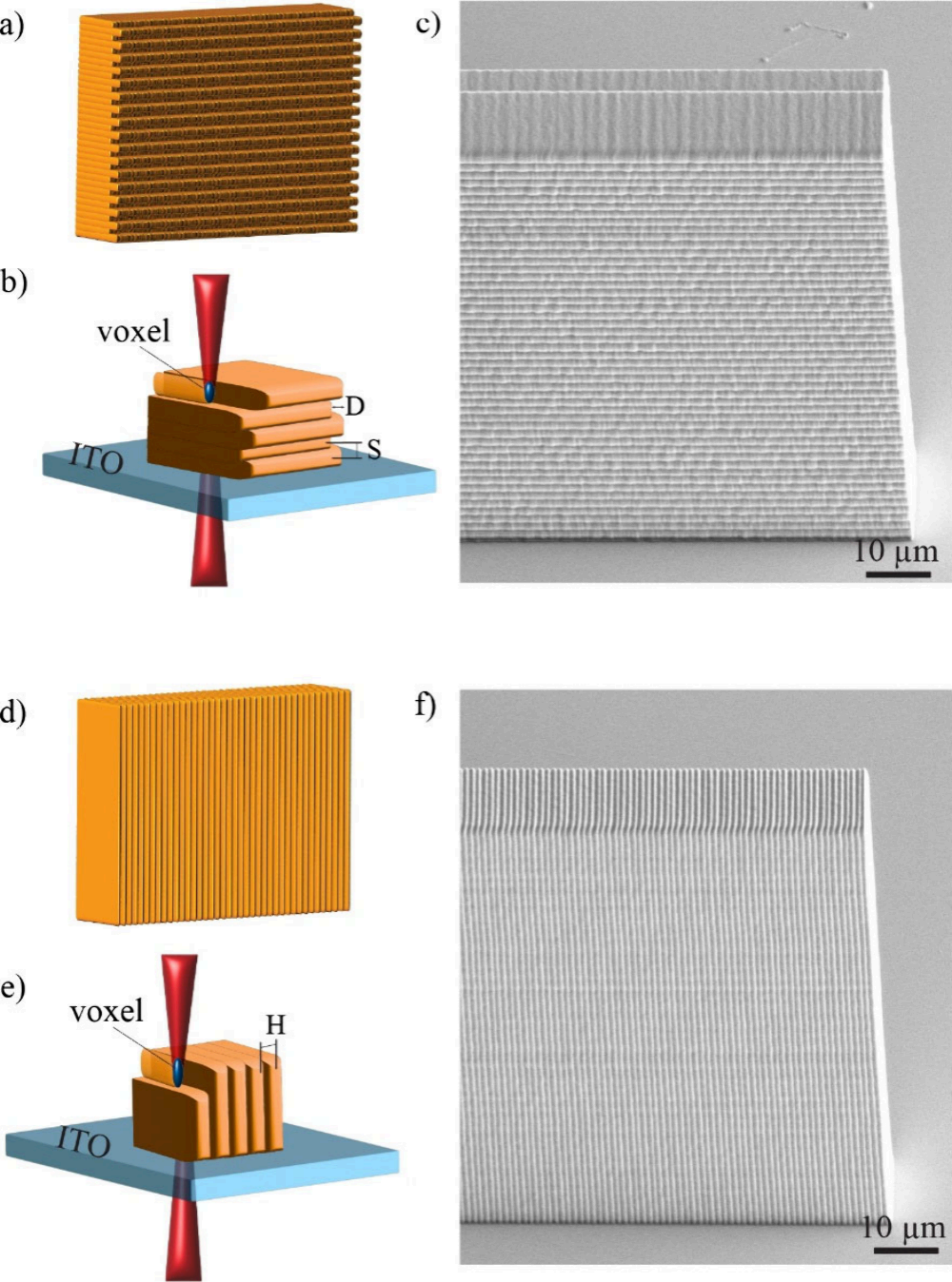
(a) Schematic representation of printing of horizontal nanogrooves. (b) Horizontal grooves are created on the face of the printed structure by offsetting the start and end point of the hatch lines in subsequent slicing layers. (c) SEM image of horizontal grooves on a vertical polymer wall. The slicing distance of 500 nm creates periodicity of ∼1010 nm. (d) Schematic representation of printing vertical grooves. (e) Vertical grooves are created by keeping the position of the end points fixed, by increasing the hatching distance and decreasing the slicing distance. (f) SEM image of vertical grooves. The hatching distance of 400 nm produces periodicity of 410 nm.

While all these achievements clearly demonstrate the power of DLW technique in engineering novel LC architectures and functionalities, the alignment of NLCs is still restricted to a plane that is also the focal plane of DLW microscope that is used for printing. To produce alignment on walls that are vertical or tilted with respect to the focal plane is still a major challenge and remains largely unexplored.^22,23^ The ability to pattern structured surfaces with high resolution in 3D would make it possible to design and fabricate advanced LC-based optical elements and tunable microphotonic devices,^24^ where precise and well-controlled molecular organization in 3D is crucial.

## 2 Results and Discussion

Here we show that DLW printing can produce well-defined planar alignment on vertical walls of polymer scaffolds and we demonstrate the feasibility of our approach. As a proof of concept, we produce a chequerboard pattern with alternating micropixels on vertical walls of rectangular microprisms, which is an array of 90° twisted nematic microdisplays with good contrast ratio.

Polymer structures are fabricated using a DLW system (Photonic Professional GT2, Nanoscribe GmbH) using a 63×, 1.43 NA objective, which has a print field of 150 μm × 150 μm and allows for DLW printing on a 127 mm × 127 mm area by stitching. A high-power femtosecond laser beam of 780 nm wavelength is moved inside a photosensitive resin, which is drop casted onto a substrate. The power of the laser is adjusted to the level, where the two-photon polymerization of the resin occurs in the focal (i.e., horizontal) plane of the objective. The cross-section of the polymerized volume in the *x*–*y* (focal) plane is ∼120 nm and ∼300 nm in the *z* direction (optical axis). An object is printed by dividing the structure into slices (i.e., horizontal layers), which are separated by a slicing distance S. Within a single slice, the polymerization voxel is moved using galvo mirrors line by line, with line separation called the hatching distance H. As one slice is completed the stage is moved vertically (i.e., along the optical axis of the objective) and the next slice is printed.

To print our structures, a small amount of photo sensitive resin is drop-casted onto a 170 μm thick glass substrate with 30 nm ITO coating (Diamond Coatings Ltd.), see the SI. We use the dip-in printing method, where the resin acts as the immersion, index matching, liquid. The glass substrate is placed above the printing objective and the space between the objective lens and the substrate is filled with the resin. The print starts at the surface of the substrate and the objective is gradually moved downward during the printing. In this way, the laser beam always passes through optically homogeneous resin, thereby preserving the high quality of the focused writing beam, eliminating aberration. The end points of the laser lines can still produce uneven edges of each slice, therefore when needed, an additional scan running all around the contour of the structure is performed after each slice is completed to ensure maximum surface smoothness (i.e., contour plot). Our aim is to create nanogrooves imprinted into vertical walls, which would be oriented either horizontally (parallel to the focal plane of the objective) or vertically (i.e., perpendicular to the focal plane).

To create horizontal nanogrooves on a vertical wall, shown in Figure 1a, the voxel is moved in a direction perpendicular to that wall and the hatching distance is decreased to the value that fuses and smoothens the two neighboring lines so as to create a single slicing layer with smooth edges. When printing the second slicing layer on top of the first one, the laser is turned-off at some distance D before the end of the first layer (Figure 1b). This creates a “depression” in the printed layers that goes in a horizontal direction, and becomes a horizontal nanogroove after the third layer is printed in the same way as the first one, and on top of the second layer, as shown in Figure 1b. Figure 1c shows SEM image of printed horizontal nanogrooves that cover the entire vertical surface of a 100 μm × 50 μm polymer block printed with high resolution resin IP-Dip2. The hatching distance of this print is H = 100 nm, the slicing distance is S = 500 nm, the edge offset of subsequent slicing layers is D = 1 μm. One can clearly see very regular and well-formed horizontal nanogrooves with the periodicity of 1010 nm in the vertical direction that cover the entire vertical surface of the polymer block uniformly.

To create vertical microgrooves on a given vertical wall, the voxel is moved in a direction perpendicular to that wall, but the hatching distance is increased to the maximum value that still ensures structural stability of the print. After the neighboring line is printed at rather large hatching distance in the same slicing layer, the edge of the printed layer will appear comb-like. When the second layer is printed on top of the first one, the slicing distance is decreased to make the print smooth in vertical direction. However, in-plane the trajectory of the voxel is kept identical, which creates another layer with comb-like edges that precisely match the first layer, as shown in Figure 1e. By repeating this print layer by layer, grooves form in a vertical direction along the vertical wall of the printed structure, because the combs are aligned with each other in adjacent layers. Figure 1f shows SEM image of vertical nanogrooves that were printed on the vertical wall of a polymer block using IP-Dip2 resin. To produce these grooves, the hatching distance was set to 400 nm, and the slicing distance was set to 100 nm, while the edge position of the beam was kept equal for all slices. This results in extremely well-defined vertical nanogrooves that cover the entire 100 μm × 50 μm vertical surface of the printed polymer block uniformly.

The surface properties of nanogrooves were explored using AFM on IP-Dip2 and IPS printed blocks measuring 5 μm in width, 50 μm in height, and 100 μm in length that were detached and placed flat onto a substrate after printing, so that the originally side surfaces of the printed blocks now face up (i.e., the overturned blocks). Two different resins, IP-Dip2 and IPS, were used to explore the effect of material properties on groove formation and LC alignment. IP-Dip2 is known for its higher resolution printing capability due to tighter polymerization threshold and mechanical stability, while IPS offers smoother surfaces and improved optical clarity, making it more suitable for applications requiring high optical quality. AFM scans of vertical grooves printed with different hatching distances are shown in Figure 2a and b. At a hatching distance of H = 100 nm, completely smooth surface was formed for both resins, with the average roughness of ∼5 nm for IP-Dip2 and ∼2 nm for IPS, (Figure 2aI and bI), indicating perfect overlap of the voxels. As the hatching distance increases, the overlap of the voxels diminishes, and a periodic vertical line-pattern emerges, as shown in Figure 2aIII and bIII. Since this pattern was repeated layer by layer, nanogrooves are formed along the vertical walls of the printed structures. At a hatching distance of H = 300 nm, shown in Figure 2a and bII, periodic nanogrooves with a periodicity of 310 nm are observed for IP-Dip2 and IPS, closely matching the set hatching distance. Due to the difference of the polymerization volume of IP-Dip2 and IPS, the groove peak-to-peak height is 40 nm for IP-Dip2 and 10 nm for IPS. Increasing the hatching distance further causes less overlap of neighboring hatch lines, resulting in mechanically unstable structures. At a hatching distance of H = 500 nm, shown in Figure 2a,b II, nanogrooves have a periodicity of 515 nm for both resins. The peak-to-peak nanogroove height is ∼240 nm for IP-Dip2 and 110 nm for IPS. For IP-Dip2, the nanogroove pattern are first observable at a hatching distance of H = 200 nm, but at H = 400 nm, the peak-to-peak height is already exceeding 100 nm, which deteriorates the optical smoothness of the surfaces. This means that height amplitude for IP-Dip2 is very sensitive to the hatching distance H. In contrast, the IPS, with its smoother polymerization volume, starts to show the nanogroove pattern at larger hatching distances.

**Figure 2:**
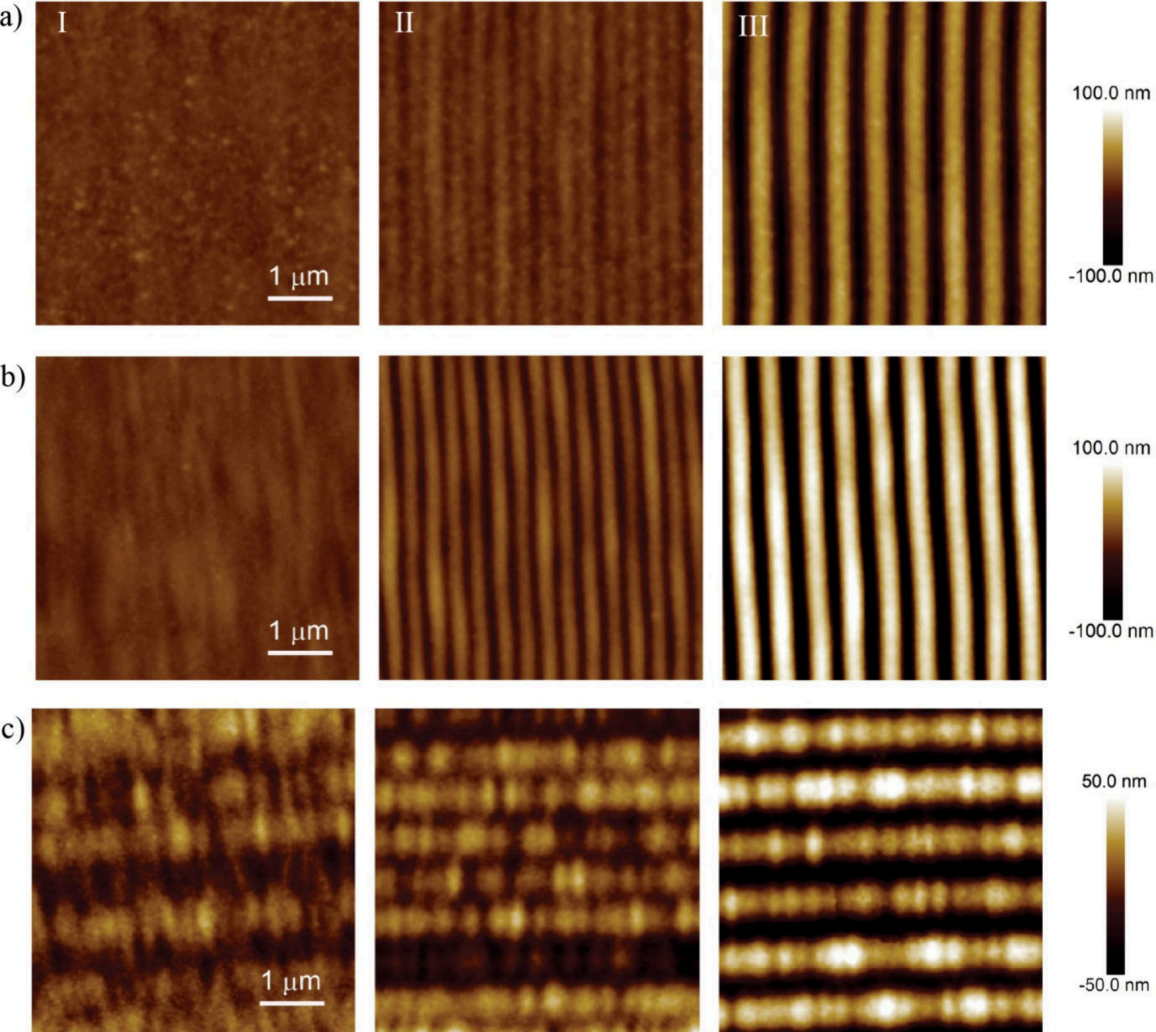
AFM images of 5 μm × 5 μm regions of the vertical walls on collapsed microblocks with vertical alignment nanogrooves printed with (a) IPS resin and (b) IP-Dip2 resin. The hatching value has been varied from 100 nm (I), 300 nm (II) to 500 nm (III). (c) Horizontal nanogrooves printed in IP Dip2, created on the vertical side wall by offsetting the hatching line start and end points. The slicing is 100 nm (I), 300 nm (II), and 400 nm (III).

Horizontal grooves on vertical surfaces of microblocks with the same dimensions were printed by offsetting the laser line end positions for adjacent slicing layers. The hatching distance was fixed to H = 100 nm to ensure smooth layers, while the slicing distance was varied from S = 100 nm to S = 500 nm to account for the high aspect ratio of the printing voxel, while stacking the slicing layers in the *z*-direction. Next, in every second printed layer the laser line end position was offset by D ∼ 1 μm. Typical AFM scans of horizontal nanogrooves for different slicing distances are shown in Figure 2c. For small slicing distances S ∼ 100 nm, a homogeneous smooth surface is observed (Figure 2cI). By increasing the slicing distance, the voxel overlap between adjacent slicing layers is significantly reduced, and the horizontal microgrooves start to emerge at around S ∼ 200 nm. For a slicing value of S ∼ 300 nm, a period of the microgroove pattern of 598 nm is observed with peak to peak height of ∼22 nm, as shown in Figure 2cII. For a slicing value of 400 nm, a stable horizontal nanogroove pattern is observed with a periodicity of 810 nm and a peak to peak height of ∼46 nm, as shown in Figure 2cIII. The periodicity of the nanogrooves corresponds to roughly doubled slicing value and the pattern is observed at slicing between 150 and 400 nm.

The grooves are uniform over the entire printing area, as shown in Figure 3a–e.

**Figure 3:**
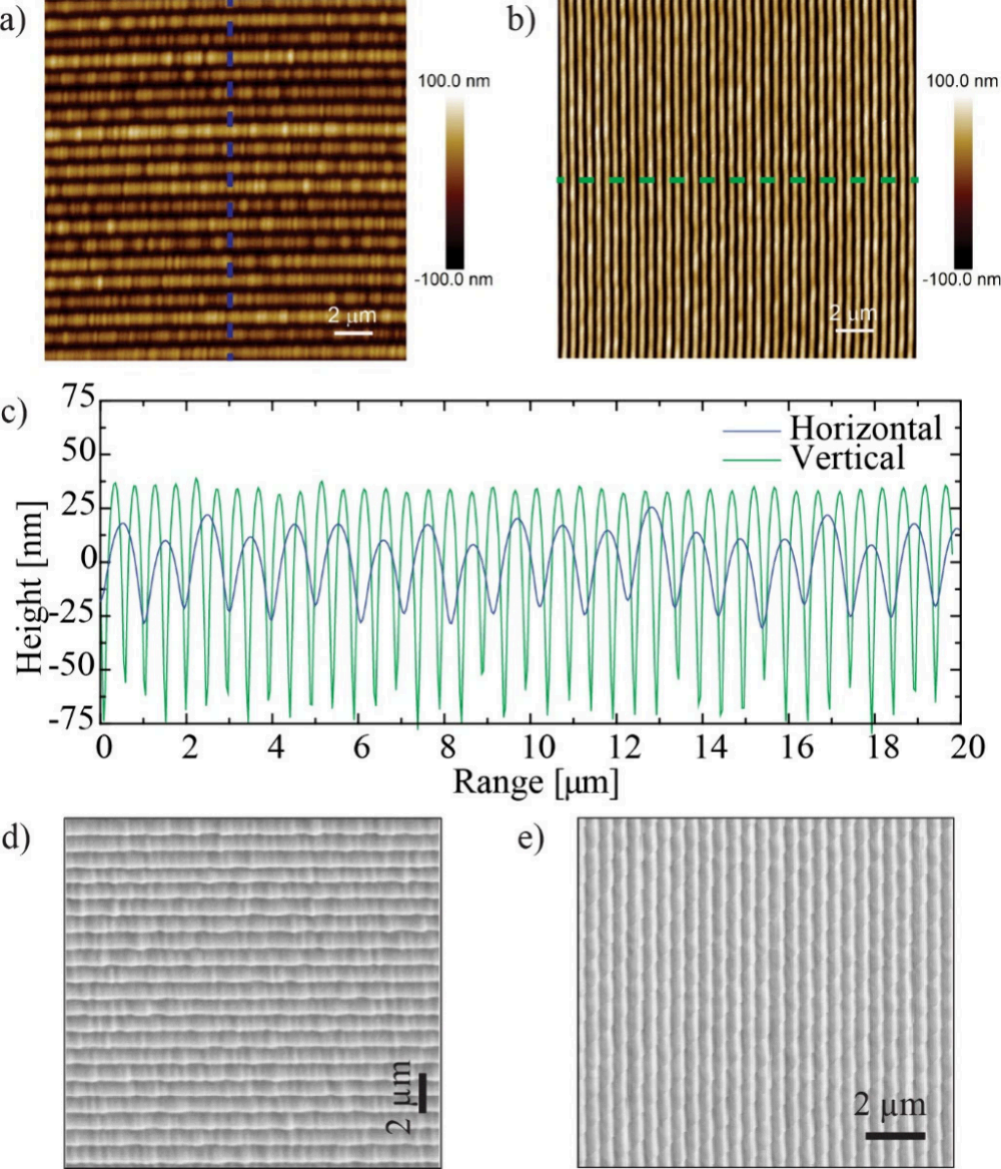
(a) AFM image of horizontal microgroove pattern fabricated with printing parametersfor IP-Dip2: S = 500 nm, H = 100 nm, offset D = 1 μm. (b) AFM image of a vertical alignment microgroove pattern on the surface made with printing parameters S = 100 nm and H = 500 nm. (c) Cross sections along the dotted lines in (b) and (c) show the range of the microgroove patterns. (d) SEM image of large area with horizontal nanogrooves. (e) SEM image of large area with vertical nanogrooves.

The optical quality of surface alignment of a NLC on printed nanogroove surfaces was characterized by measuring the optical contrast for light passing through a 90° twisted NLC cell made from an overturned microblock on one side and a rubbed (i.e., aligned) polyimide on glass substrate filled with NLC. When the nanogrooves and the polyimide rubbing directions are set perpendicular to each other, a 90° twisted NLC structure is created between the two surfaces. If a linearly polarized light is sent through such structure, with input polarization parallel (or perpendicular) to the NLC molecules on the entry surface, light polarization will be guided by the twisted structure and will be rotated for 90° with respect to the input polarization. The 90° twisted NLC cell will appear dark between parallel polarizers and bright between crossed polarizers.

The 90° twisted cell was filled with a 5CB NLC and the white light passing through the cell was analyzed at different print parameters, as presented in Figure 4a,b. For vertical nanogrooves with a hatching distance of 300 nm (Figure 4a1,2) the cell appears bright between crossed polarizers, see the graph in Figure 4c, which indicates 90° twist of the LC. However, for hatching distance of 100 nm, the cell appears dark between crossed polarizers, which indicates zero twist of the NLC. This means there are no alignment grooves due to the printing process, but the NLC is aligned solely by the orienting action of the polyimide surface. As we increase the hatching distance, we notice the gradual darkening of the cell between parallel polarizers and increase of the optical contrast, as shown in Figure 4c. At hatching values of 300 nm and above, the optical contrast is *I*_crossed_/*I*_parallel_ ∼ 7, where *I*_crossed_ is the cell transmission intensity between crossed polarizers and *I*_parallel_ is the cell transmission intensity between parallel polarizers.

**Figure 4:**
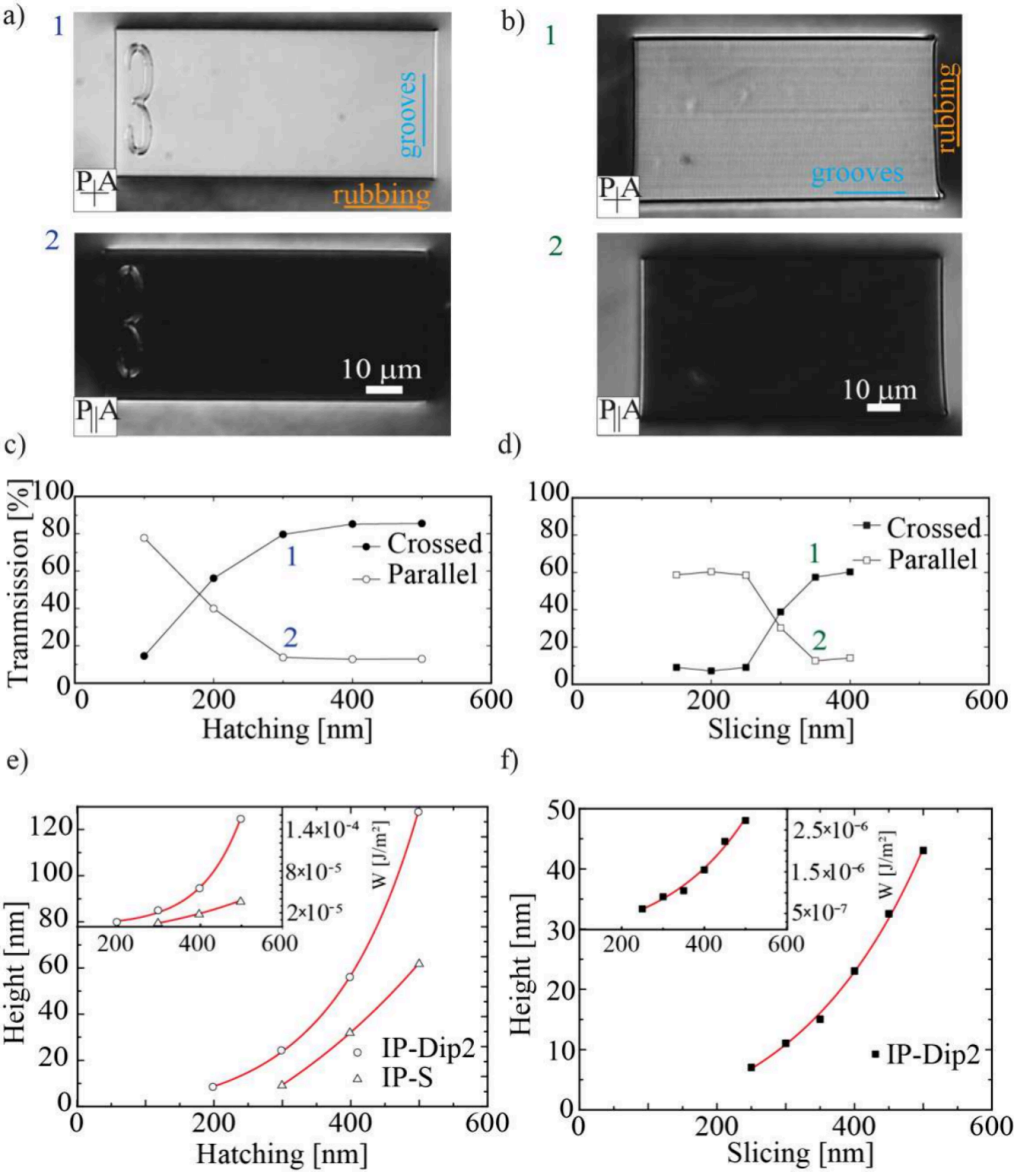
Optical quality of 90° twisted nematic cells made of one substrate with side-up DLW printed nanogrooves and the second substrate made of standard oriented polyimide layer on glass. (a) Visual appearance of 90° twisted NLC cells with vertical printed microgrooves between crossed (1) and parallel (2) polarizers. The hatching distance is H = 300 nm and the slicing distance is S = 100 nm. (b) Visual appearance of 90° twisted NLC cells with horizontal printed microgrooves between crossed (1) and parallel (2) polarizers. The hatching distance is H = 100 nm and the slicing distance is S = 350 nm. (c) Light transmission measurement through cells with vertical nanogrooves measured between parallel and crossed polarizers. (d) Transmission of light through cells with horizontal nanogrooves under the same conditions. The decrease in intensity for parallel polarizers and corresponding increase for crossed polarizers indicate the presence of a nematic twist. (e) The amplitude of vertical nanogrooves as a function of hatching distance. The inset shows the calculated azimuthal anchoring energy as a function of hatching distance H. (f) The amplitude of horizontal nanogrooves as a function of slicing distance S. The inset shows the calculated azimuthal anchoring energy as a function of S. The red curves in (e) and (f) are exponential fitting functions of the form *y*(*x*) = *y*_*o*_ + *A*·*e* ^*c*·*x*^.

For horizontal nanogrooves (Figure 4b1,2), we observe no twist of the NLC for slicing values of 100–250 nm, thus the transmitted intensity trough parallel polarizers is high. At higher slicing values, the anchoring of NLC molecules on the nanogrooves is strong enough to enforce a twist in the NLC between an overturned microblock and the polyimide surface. We observe a contrast of *I*_crossed_/*I*_parallel_ ∼ 5 at slicing distance of 400 nm (Figure 4d).

Finally, we measured the in-plane azimuthal anchoring strength of 5CB on an overturned microblock for both vertical and horizontal microgrooves printed in IPS and IP-Dip2 resins. We use an optical method,^25^ which measures the total twist angle Φ_*t*_ of the NLC in the 90° TN cell, where the preferential directions of molecular alignment on each surface are perpendicular to each other. Namely, the actual twist angle of the NLC is always smaller than 90°, because the elastic distortion of the twisted NLC prefers the NLC untwisted. Using this method, we determined the azimuthal anchoring strengths on standard rubbed polyimide (PI), which is 4 × 10^–5^ J/m^2^, in agreement with literature.^2^ For vertically aligned nanogrooves on IPS with a hatching distance of H = 500 nm, the azimuthal anchoring energy is 4 × 10^–6^ J/m^2^. The corresponding groove amplitude was 56 nm and the periodicity 515 nm, which yields an estimated anchoring strength of 3.4 × 10^–5^ J/m^2^ according to the model.^1^ The measured azimuthal anchoring strength in IP-Dip2 was 1 × 10^–5^ J/m^2^ for nano-ogrooves with H = 500 nm. The corresponding groove amplitude of 120 and 515 nm periodicity yields an estimated anchoring strength of 1.5 × 10^–4^ J/m^2^ from Berreman’s model. The observed deviations between the model predictions and the measured values can be attributed to the limitations and assumptions of the Berreman model, which is valid only for small-amplitude sinusoidal surface modulations.

The corresponding azimuthal anchoring strengths, calculated from the Berreman model^1^ as a function of hatching distance H for vertical (Figure 4e) and horizontal (Figure 4f) nanogrooves for two different resins, are shown in the insets. It is clear that the azimuthal anchoring strengths of NLC on nanogrooves printed by DLW are not only comparable to standard PI anchoring strengths, but can be tailored by simply changing the hatching distance in the interval ∼10^–5^ to 10^–7^ J/m^2^.

In this Article, we show in Figure 5 how the novel technique of DLW printing the LC alignment layers could be used to produce LC-based microphotonic devices with novel architecture and functionality. Figure 5a is a SEM image of a DLW printed polymer scaffold consisting of two right-angle prisms, facing each other at a gap separation of 10 μm. The gap is part of a closed volume–a container that incorporates two semicylindrical side containers. On the facing edges of the prisms, two, 2 μm thin rectangular plates with a height of 70 μm were printed. On one of the vertical surfaces of these rectangular plates, a 12 μm × 17 μm checkerboard pattern of periodic nanogrooves was printed to align the NLC in a chequerboard fashion (Figure 5b), while the other surface has vertical alignment grooves with periodicity of 400 nm imprinted, thus forming array of alternating twisted and nontwisted NLC areas. Using a microinjector, the cavity between the prisms is filled with 5CB NLC. To visualize the liquid crystal (LC) alignment within the cavity between the two prisms, the structure was observed through the prisms themselves under a polarized optical microscope, where the light passes through the structure in direction of the yellow arrow. The very good quality of surface alignment of nematic liquid crystals (NLC) on printed nanogrooved surfaces is seen from the polarized optical images of checkerboard pattern in Figure 5c,d.

**Figure 5:**
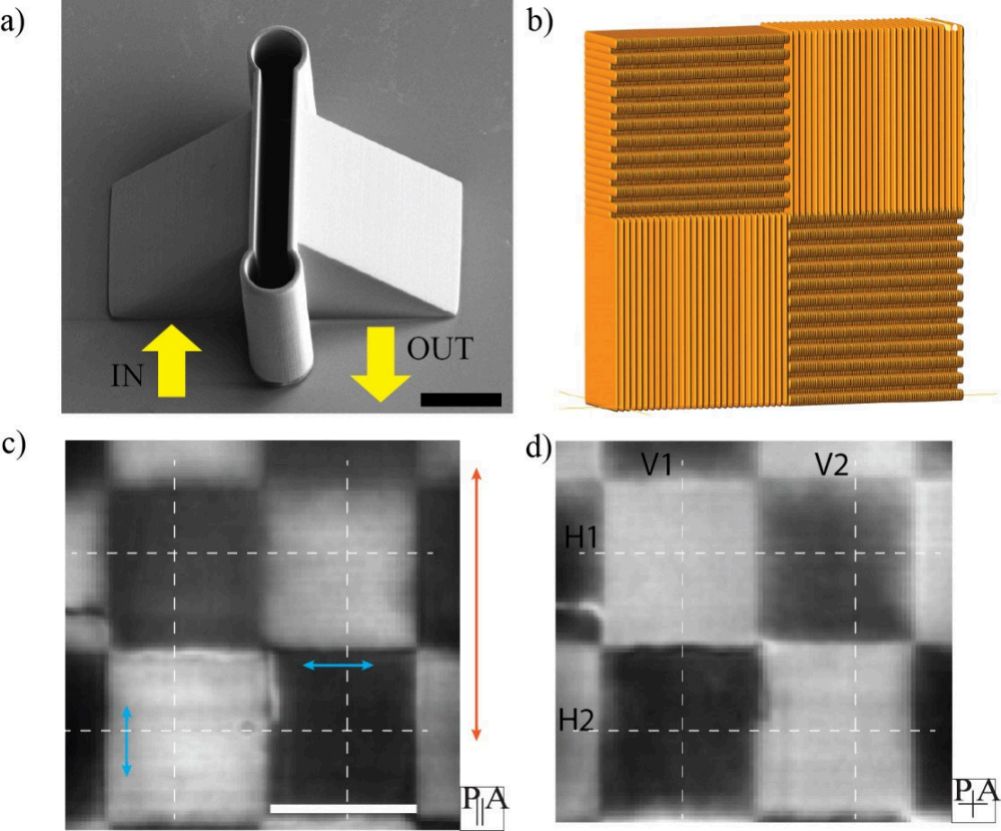
(a) SEM image of the polymer microprisms with a base size of 50 μm that were printed using a 63× objective and IP-Dip2 resin. Light path is indicated by the yellow arrows. Scale bar 20 μm. (b) Schematic representation of the checkerboard pattern of alignment grooves on the prism surface. (c) Image of a checkerboard pattern taken with an oil immersion 100× objective through the microprism, using parallel polarizers. Dark fields represent 90° twisted pixels, bright fields represent homogeneous nematic 5CB. Scale bar 12 μm. Blue arrows show the checkerboard groove orientation while the orange arrow shows the groove orientation on the opposing prism face. (d) The same area as in (c), but viewed between crossed polarizers. Pixel size is 12.5 μm × 17.5 μm. The dashed lines in (c) and (d) mark the location where cross sections were analyzed.

To further quantify the homogeneity of the alignment, the images shown in Figure 5c and d were merged to obtain the polarization contrast, calculated as (*I*_parallel_ – *I*_crossed_)/(*I*_parallel_ + *I*_crossed_). The resulting scaled image is presented in Figure 6a, where regions of different alignment are clearly distinguished by their relative transmission intensity. From the same data set, horizontal and vertical cross sections along the lines marked in Figure 5c and d were extracted and are shown in Figure 6b. These cross sections directly visualize the local variations in contrast between the two orthogonal polarizations. The difference in the flatness of the profiles reveals that the alignment in the horizontal direction is less homogeneous, which we attribute to partial flow-induced alignment during the filling of nematic liquid crystal into the prism cavity. Despite this, a transmission contrast of up to ∼6 is observed, which confirms the high efficiency of the printed nanogrooves in inducing strong surface anchoring, while also highlighting the sensitivity of the method to subtle inhomogeneities in LC orientation.

**Figure 6:**
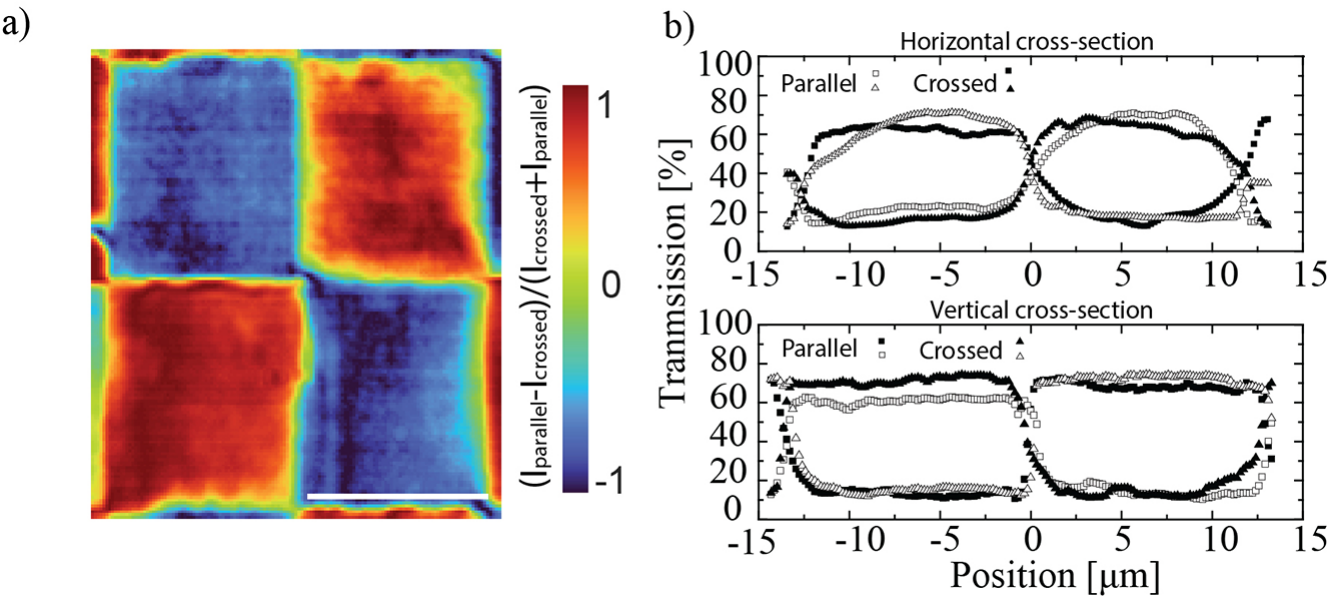
(a) Scaled image of the polarization contrast (*I*_parallel_ – *I*_crossed_)/(*I*_parallel_ + *I*_crossed_). (b) Horizontal and vertical cross sections at both polarizations are observed. The difference in the flatness of the profiles indicate that the alignment in the horizontal direction is less homogeneous, which might be due to flow alignment caused by the filling of the prisms. One can observe a contrast of transmission of up to 6.

## 3 Conclusion

In conclusion, we demonstrated novel method of aligning NLCs along flat vertical surfaces of polymer scaffolds that is based on DLW printed nanogrooves. The method is robust, reliable, repeatable, and can be well controlled. The advantages of this new method are at least 2-fold. First, we are able to print large areas of well-defined patterns of nanogrooves, and, second, we are able to control the azimuthal anchoring strength by controlling and adjusting the amplitude and the periodicity of the microgrooves. We can therefore manipulate the LC orientation with high accuracy, enabling efficient control over the optical functionality of the DLW printed system. While the method has been applied here to create two typical types of alignment, one could envision extensions far beyond these limited cases. For instance, complex patterns such as spirals, radial gratings, or q-plates with topological defects may become feasible by exploiting the anisotropy of the voxel or by dynamically controlling the exposure profile during printing. The voxel itself naturally narrows at the ends of print lines due to mechanic inertia of the moving mirrors and laser turn-off dynamics, which has already been exploited in proprietary grayscale lithography techniques developed for two-photon polymerization. Future improvements may also target more isotropic voxel shapes and reduced voxel size by employing adaptive optics such as spatial light modulators (SLMs) to correct the point spread function (PSF), or by engineering the resist chemistry to improve threshold sharpness and nonlinearity. Inspired by STED microscopy, subdiffraction-limited DLW has already demonstrated with lateral resolutions below 50 nm and axial resolution below 150 nm.^26^ Alternatively, tailored photochemistry using two-step polymerization or radical quenching has achieved feature sizes down to 30–80 nm.^27^ These emerging techniques could eventually enable full 3D alignment patterns with programmable complexity and sub-100 nm feature sizes. Finally, DLW printed alignment patterns could be used as a master to produce replicas by soft nanoimprint lithography, preserving LC alignment.^28^

Printing of alignment nanogrooves on tilted or curved surfaces remains a challenge, but we are confident that such patterns can be realized with appropriate modifications to the printing strategy. Overall, this technique offers a highly versatile platform for developing complex liquid crystal alignment architectures for microphotonic elements.

## Supplementary Material



## Data Availability

The data that support the findings of this Article are openly available.^29^

## Notes

Sample preparation for DLW printing is described in detail. Scanning electron imaging and atomic force microscope imaging of DLW printed structures is presented in detail. Characterization of aligning properties of DLW printed nanogratings is presented, including measurements of the azimuthal surface anchoring strength of printed substrates and measurements of optical contrast (PDF)
