# Supplementary material for: Three-Dimensional Planar Alignment of Nematic Liquid Crystal by Direct Laser Writing of Nanogratings
Source: ACS Photonics. 2025 Oct 16;12(11):5970–7. doi: 10.1021/acsphotonics.5c01342 (PMC12636081; doi:10.1021/acsphotonics.5c01342)
Supplement: Supplementary file 1 [file ph5c01342_si_001.pdf]

Three-dimensional planar alignment of nematic liquid crystal by direct laser writing of nano-gratings

*Uroš Jagodič[1], Jaka Pišljar[1,2], Andreja Jelen[1], Miha Škarabot[1,2], Igor Muševič[1,2]**

[1]Condensed Matter Department, Jožef Stefan Institute, Ljubljana SI-1000, Slovenia

[2]Faculty of Mathematics and Physics, University of Ljubljana, Ljubljana SI-1000, Slovenia

Supporting Information.

Sample preparation:

The substrates are thoroughly cleaned with lint free cloths, isopropanol and acetone, after which they are sonicated for 15 min at 35°C in detergent (Hellmanex II, Hellma Analytics) water baths. After sonication, the substrates are thoroughly rinsed with pure DI water and dried in an isopropanol vapour degreaser. To enhance the adhesion of printed structures, the substrates are placed into a UV plasma for 5min. Immediately after the plasma treatment, a drop of IPS/IP-Dip2 resin (Nanoscribe GmbH, Germany) is placed onto the substrate.

SEM and AFM imaging:

Printed structures with horizontal and vertical alignment grooves on vertical walls were imaged using SEM. The structures were coated with a thin 5 nm layer of gold/palladium. After the coating the imaging was performed by Thermo Fisher Verios 4G HP Schottky field-emission SEM, using secondary electrons (SE). The side planes of the printed structures were imaged by tilting the substrate by 30°. Additionally thin blocks with different alignment layers on their sides were printed and then manually overturned onto the substrate using a precise manipulator of a microinjector (FemtoJet 4i, Eppendorf). The surfaces of some of the overturned structures were imaged using Atomic Force Microscope (AFM, Nanoscope IIIa, Digital Instruments, USA) in tapping mode.

Characterization of alignment properties:

In addition to surface characterization by SEM and AFM, two different optical measurements were performed to characterize the quality of surface alignment of a NLC in contact with DLW printed micro-grooves:

(i) The strength of azimuthal surface anchoring of a NLC on printed microgrooves was measured in cells that were assembled from one glass slide with overturned structures and another glass slide that was covered with a 20 nm layer spin-coated Polyimide (PI 5291 Nissan, Japan) and uni-directionally rubbed with a velvet cloth. The rubbing direction of the PI sets the direction of orientation of NLC on that surface and this direction was set at 90° with respect to the direction of printed micro-grooves on the adjacent surface. The cell thickness was determined by 15 μm glass spacers, and the cell was filled with 5CB LC by capillary action. The cells were observed under an upright polarising microscope (Nikon Ecllipse e600 POL), equipped with Flir BFS-U3-50S5C camera (capable of imaging at 16-bit monochrome) and illuminated using CoolLED pe-100 source and a 10 nm bandpass filter cantered at 550 nm. The azimuthal anchoring strength was determined by measuring the local twist angle in a twist nematic cell.[23] Briefly, for an infinite azimuthal surface anchoring strength, the total helical twist of the NLC would be exactly 90°. However, the elasticity of the twisted nematic produces a counter-torque that forces the molecules close to the surface to point slightly off the direction dictated by the surface. This means that the actual twisting angle Φ_{TN} is less than 90° for finite values of the azimuthal surface anchoring energies. This makes it possible to measure the azimuthal anchoring strength by measuring the actual twist of the NLC in 90° twisted cells. The twist of the NLC is measured optically by rotating the

polarizer and analyser and determining their positions that give a global minimum light transmission.

(ii) To characterize the optical quality of the alignment on vertical walls, we printed a pair of prisms positioned to face each other with the vertical walls. On the surfaces facing each other alignment grooves were printed and the separation between the walls was 8 μm. Next, using the micro-injector and a micropipette with 1 μm outer diameter, the space between the prisms was filled with 5CB NLC, which aligned along the printed microgrooves on these vertical walls of the micro prisms. In this way, a micro LCD was produced between the two micro-prisms, which could be observed using an inverted polarizing microscope Nikon Ti-u equipped with CoolLED pE-300 illumination source and a FLIR BFS-U3-50S5C camera. The images obtained under parallel and crossed polarizers were imported into MATLAB. A Gaussian illumination background was estimated from the mean of the two images by selecting the top 50% of the brightest pixels. This background was subtracted from both images, and the results were normalized to ensure a comparable intensity scale. A region of interest (ROI) was then selected over two adjacent alignment squares. To assess the uniformity of the alignment, the polarization contrast was calculated as $(I_{parallel} - I_{crossed})/(I_{parallel} + I_{crossed})$ and visualized as a rescaled image. Additionally, vertical and horizontal intensity cross-sections were plotted to illustrate the optical contrast between the images taken under crossed and parallel polarizations.

Corresponding Author

*Igor Muševič, Condensed Matter Department, J. Stefan Institute, Jamova 39, SI-1000 Ljubljana, Slovenia, Email: igor.musevic@ijs.si

Author Contributions

U.J. and I.M. conceived and supervised the research. U.J. developed the printing code, printed the objects, performed optical experiments and analyzed the data. J.P helped with optical experiments. A.J. performed SEM imaging. M.Š. performed the AFM measurements and analyzed the data. U.J. and I.M. wrote the manuscript. All authors have given approval to the final version of the manuscript.

For Table of Contents Use Only

Three-dimensional planar alignment of nematic liquid crystal by direct laser writing of nano-gratings

Uroš Jagodič, Jaka Pišljar, Andreja Jelen, Miha Škarabot, Igor Muševič

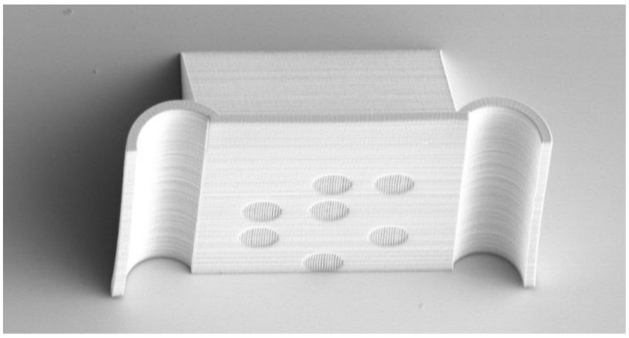

This Scanning Electron Microscope image shows a face of a 90° micro-prism that was DLW printed on glass. This vertical surface was printed in a way to produce horizontal nano-grooves throughout the surface that provide a good quality alignment of a nematic liquid crystal when in contact with this surface. One can see the logo of Josef Stefan Institute that was produced by printing vertical nano-grooves in designated circular patches.
